# Hydrodissection and femtosecond laser assisted surgery in posterior polar cataract

**DOI:** 10.1007/s10792-024-03286-4

**Published:** 2024-09-05

**Authors:** Ehud I. Assia, Arie Y. Nemet

**Affiliations:** 1 Ein-Tal Eye Center, 15 Habarzel St, 6971021 Tel Aviv, Israel; 2 Department of Ophthalmology, Meir Medical Center, Kfar Saba, Israel; 3 School of Medicine, Tel Aviv University, Tel Aviv, Israel

**Keywords:** Posterior polar cataract, Posterior capsule rupture, Hydrodissection, Femtosecond laser cataract surgery

## Abstract

**Purpose:**

To present our clinical experience using femtosecond laser-assisted cataract surgery (FLACS) and cortical cleavage hydrodissection in eyes with posterior polar cataract.

**Methods:**

Medical records of consecutive10 eyes of 6 patients with clinical diagnosis of posterior polar cataract (PPC), were retrospectively reviewed. All surgeries were done by using femtosecond laser-assisted cataract surgery. In all cases careful hydrodissection was done to separate the lens material from the posterior capsule.

**Results:**

There were 3 males and 3 females, ages 39–73 years (average 52.5 years), two of them were implanted with toric lenses. In all eyes hydrodissection was successfully performed and the lens material was separated from the lens capsule. The posterior capsule remained intact during nucleus removal in all cases. In one eye the posterior capsule broke during cortical cleaning and the tear was converted to posterior capsulorhexis (PCCC). No postoperative complications were recorded during follow-up in all eyes.

**Conclusions:**

Hydrodissection can be safely performed in combination (but not exclusively) with FLACS, in eyes with posterior polar cataract with no evidence of a preexisting posterior capsule rent. Hydrodissection is regarded by most surgeons as contraindicated in these eyes however apparently it is more gentile to the capsule than any other surgical maneuver and allows clean and efficient separation of the lens material from the thinned posterior capsule. Femtosecond laser capsulotomy and lens fragmentation is effective and may further assist surgery by pneumo-separation of the lens material. Anterior chamber maintainer may further aid to the stability of the chamber and safety of surgery.

**Supplementary Information:**

The online version contains supplementary material available at 10.1007/s10792-024-03286-4.

## Introduction

Posterior polar cataract (PPC) is a type of developmental cataract with a distinct morphology, characterized by a white, multiple concentric layers, well-defined, distinctive discoid opacity located on or in front of the central posterior capsule (PC) [1–3].

PPC have always been a surgical challenge because of the adherence of lenticular opacity to the thin and fragile posterior capsule. This is associated with high propensity for posterior capsule rupture during surgery. Over the years, several technical modifications have been suggested to enhance safety and reduce posterior capsule rupture (PCR) rates in eyes with polar cataracts [3–5].

In general, the incidence of PCR in routine cataract surgery is less than 1%, [6] while the incidence of PCR in eyes with PPC is reported in older studies as high as 26–36% [5, 7]. More recent studies found lower rate of PCR. Hayashi et al. [8] have reported PCR in 7.1% of 28 eyes , Haripriya et al. had 12.5% PCR in their cases [9], Malhotra showed a lower PCR rate of 7.6%. [10] and one study had a low rate of 4% using femtosecond lasers in PPC surgery [11].

To prevent this dreaded complication, many modifications were proposed. Fine et al. in 2003 advocated minimal hydrodissection in multiple quadrants combined with hydrodelineation to separate the nucleus from the cortex [12]. Allen et al. used viscodissection to separate only the peripheral cortex from the capsule [2]. To date, there is no consensus on a single surgical strategy that can eliminate the occurrence of PCR in a PPC [3]. Cortical cleaving hydrodissection is conventionally considered by many surgeons as a relative or absolute contraindication in posterior polar cataracts as the hydraulic pressure can cause or extend a PCR [1, 7]. Nuclear rotation is also considered contraindicated as the margins of the opacity could act as a trephine and cause a posterior capsular tear [1, 2].

We herein describe our clinical experience using a controlled hydrodissection and occasionally nuclear rotation in eyes with typical posterior polar cataract operated using femtosecond laser.

## Methods

### Patients

This was a retrospective, non-randomized, interventional consecutive case series. The medical charts of eyes with posterior polar cataract, operated by one surgeon (EIA) were retrospectively reviewed.

All patients had preoperative detailed ophthalmological examination including recording of uncorrected distance visual acuity (UDVA), corrected distance visual acuity (CDVA), intraocular pressure measurement, slit‑lamp biomicroscopy, and dilated fundus examination. Pupillary retroillumination was done in every case to look for pre‑existing capsular breaks. In all cases femtosecond laser was utilized using routine parameters.

All patients were informed regarding the risks of surgery and signed an informed consent. The IRB of the Meir Medical Centre Ethics Committee waived the requirement for informed consent from the study subjects.

### Surgical technique

One experienced surgeon (EIA) performed all the surgeries at the Ein-Tal Eye Center, Tel-Aviv. In no case was a preexisting posterior capsule tear seen or suspected.

In cases of toric intraocular lens (TIOL) implantation, ink dots were marked preoperatively, at the slit lamp, at the limbus, while the patient’s head was positioned vertically.

**Fig. 1:**
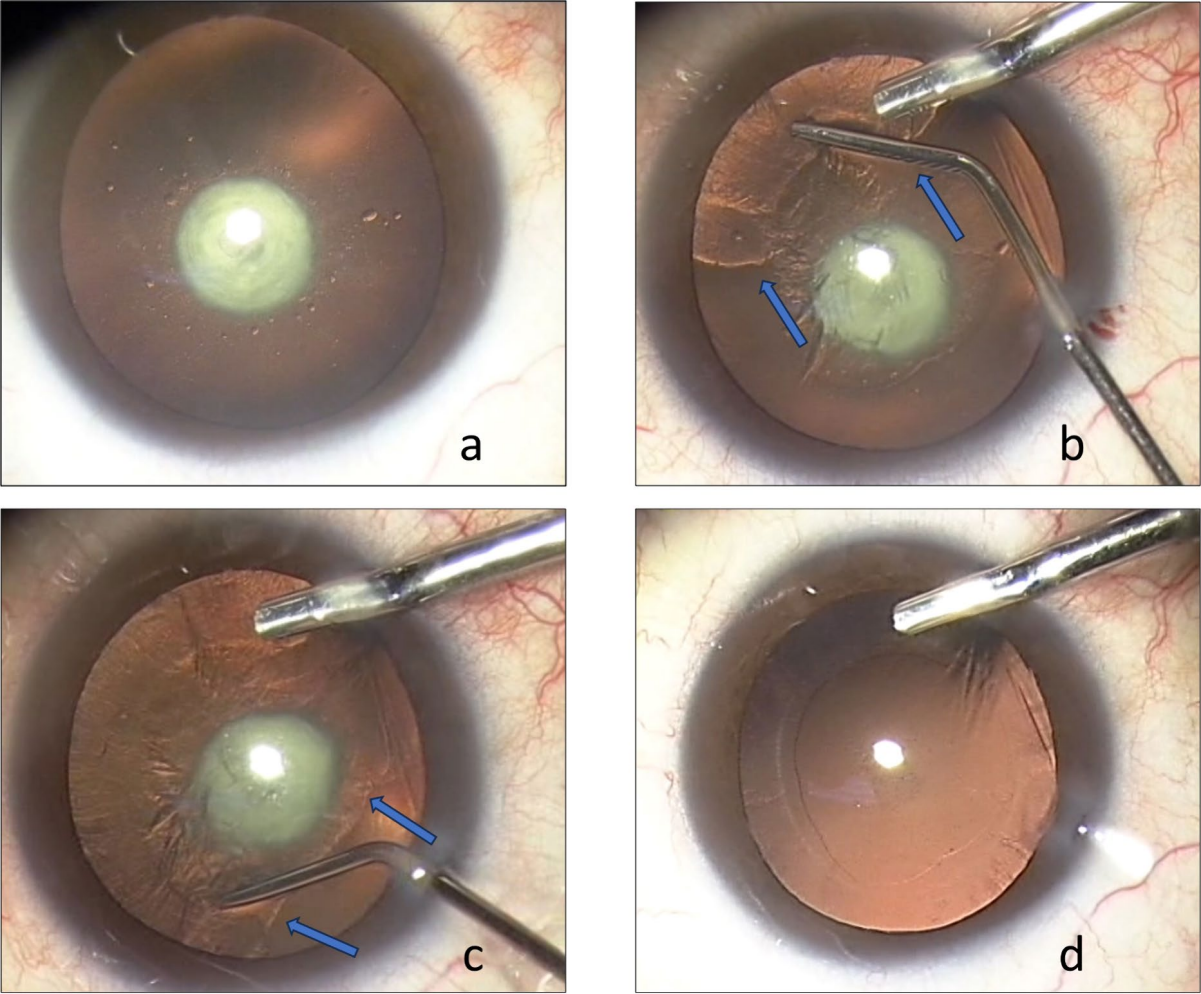
Hydrodissection in an eye with posterior polar cataract (case done manually, not included in this study) **a** A denses central posterior polar cataract. **b** Following ACCC, hydrodissection is done gently and slowly, The water wavefront, indicated by the 2 arrows, has not yet reach the lens' posterior pole. **c** Hydrodissection is carefully continued. The waterfront ( arrows) now transpassed the posterior pole, separating the lens material from the capsule. **d** Posterior capsule in intact after complete cleaning of the cortical material

The surgical technique utilized in the present set of cases was based on previous experience of hydrodissection in a many similar cases of polar cataracts using manual capsulectomy and lens fragmentation (Fig. 1 a–d).

In the present study all patients underwent a 5.0 mm femtosecond laser anterior capsulotomy and a sextant cleavage of the nucleus to 6 pieces using the manufacturer standard parameters (Catalys Precision Laser System, Johnson & Johnson). In the 2 eyes with toric IOLs corneal marks of the desired IOL position were also done using the femtosecond laser.

**Fig. 2:**
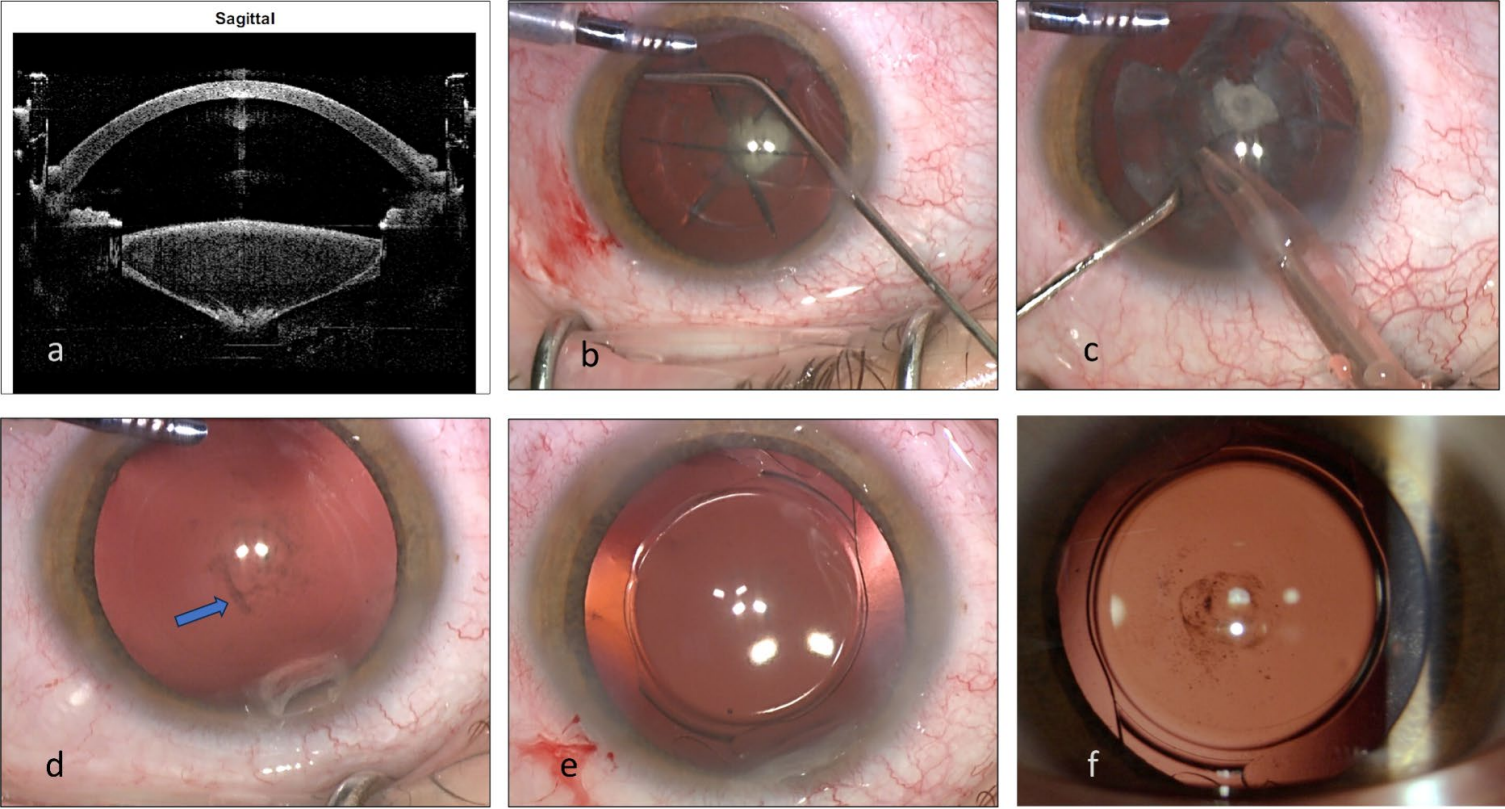
Hydrodissection in an eye with posterior polar cataract using FLACS. **a** Sagittal view by the integrated OCT of the femtosecond laser machine. A posterior polar cataract creates a posterior bulge of the lens capsule and material. There is no clear demarcation of the posterior capsule however, there is no evidence of capsular break or discontinuity. **b** Hydrodissection is carefully done between the anterior capsule and the lens material. **c** Phacoemulsification of the lens material. The posterior white plaque is separated from the capsule and flipped anteriorly. **d** After removal of the lens material the posterior capsule is intact. Pigmented dots are seen on the posterior capsule. A small localized scar, adherent to the capsule (arrow), was surgically removed using intraocular forceps leaving the posterior capsule intact. **e** PC-IOL is secured within the intact capsular bag. **f** One month post operatively the posterior stippling is seen by retroilumination however the visual acuity was not affected

The integrated optical coherence tomography (OCT) of the femtosecond laser was used to evaluate and visualize the integrity of the posterior capsule (Fig. 2a). After topical anesthesia, 2–3 side ports clear corneal incisions were made, and intracameral mixture of lignocaine and adrenaline were injected, followed by insertion of anterior chamber maintainer.

A 2.4-mm, clear corneal incision was made superiorly (at 110°). A carefull hydrodissection with Blumenthal canula (BVI) was done at three separate locations (Fig. 2b). The fluid wave was seen passing behind the lens material separating and lifting the central opacity from the posterior capsule. When a resistance to fluid passage was suspected the hydrodissection was aborted and performed at another location. Phacoemulsification (Centurion, Alcon or Signature Pro, Johnson &Johnson) was done using routine parameters of the surgeon's preference (Fig. 2c). The performance of the 2 machine was generally similar and fluidics during phacoemulsification was not affected by the gravitational pressure provided by the ACM, which was lower than the pressure provided by the machines.

Following removal of the nucleus and epinucleus, aspiration of the remaining posterior epinucleus and cortex was performed by manual aspiration using a 0.4 mm pore aspiration cannula and a continuous irrigation via the anterior chamber maintainer (Fig. 2d). A foldable IOL was implanted into the capsular bag in all cases (Fig. 2e). Typically, tiny dots were often seen on the central posterior capsule and removed by waterjet irrigation and aspiration, however occasionally the surgeon opted not to risk the integrity of the thinned capsule and treat the capsule opacification by ND:YAG laser at a later time (Fig. 2f). In two eyes a toric IOL was inserted into the capsular bag and rotated to its premarked final desired axis. Follow up visits were done a 1 day, 1 week and 1 month postoperatively.

## Results

In total, 10 eyes of 6 patients with clinically diagnosed dense posterior polar cataract (PPC) were operated between February 2020 and August 2023. There were 3 females and 3 males, with an average age of 52.5 (39–73) years. In 4 patients the PPC was operated bilaterally, and in 2 patients only 1 eye was operated. In 2 cases a toric lens was implanted. In all 10 eyes anterior capsulotomy and a sextant nucleus fragmentation were performed using femtosecond laser. The anterior segment OCT of the femtosecond laser further confirmed the clinical diagnosis of posterior polar cataract. In some cases the posterior capsule was embedded within the lens hyperreflective opacity, however there was no evidence of capsular discontinuity (i.e. rupture) in any of the eyes. Hydrodissection was successfully completed in all cases, even in cases in which the fluid wave did not pass the posterior pole on the first attempt. The typical air bubbles generated by the laser were seen along the nucleus cuts, as well as behind the lens. This often resulted in partial "pneumo-separation" of the lens material from the capsule thus assisted in the hydroseparation during hydrodissection. In none of the eyes did hydrodissection result in tear of the central posterior capsule.

Lens removal was usually completed with high vacuum and low energy settings since the patients were relatively young and the lens nuclei were typically soft. In all cases a dense central white opacity, often times presented as a well demarcated white disc, was seen and surgically removed. In 4 eyes multiple tiny dots remained adherent to the capsule and in 1 case a fibrotic scar was firmly adherent to the capsule. The capsule was cleaned as much as practical and the scar was surgically peeled off, however some opacities occasionally remained and treated Nd:YAG laser capsulotomy after a few months.

In one case the removal of the nucleus and aspiration of the cortical material were successfully completed without tearing of the posterior capsule, However, some residual tiny dots were seen on the capsule. A gentle water-jet cleaning using the hydrodissection cannula ended up with a small localized tear in the capsule. Posterior continuous curvilinear capsulorhexis (PCCC) was performed using a capsulorhexis forceps and the IOL was implanted in-the-bag.

Postoperatively, in all eyes the PC-IOL were located within the capsular bags, central and stable and the visual acuity improved.

## Discussion

Posterior polar cataract (PPC) presents a special challenge to the cataract surgeon due to its high risk of posterior capsule rupture (PCR), vitreous loss, and even nucleus drop during cataract surgery, which can occur because of capsular weakness, pre-existing breaks or tight adherence of lens opacity and the posterior capsule [13]. Hydrodissection and nucleus rotation is considered a taboo by most surgeons in posterior polar cataract extraction [14].

During phacoemulsification, PCR often occurs during the removal of the epinucleus [7] or the posterior polar opacity [5]. Extreme care has been suggested not to overpressurize the anterior chamber or capsular bag as cortico‑cleaving hydrodissection can lead to hydraulic rupture and PCR [12].

In conventional hydrodelineation, the cannula penetrates within the lens substance, not around it. It causes the fluid to traverse from outside to inside and dissect the layers of the lens, leaving a layer of at least the epinucleus attached to the posterior capsule. These techniques reduce the risk of PCR at the time of nucleus removal yet it does not prevent capsule rupture at later stages of lens removal.

Fine et al. also performed a minimal hydrodissection and hydrodelineation in multiple quadrants injecting tiny quantities of fluid gently, so that the fluid wave is not allowed to spread across the entire posterior capsule [4]. Some authors advocate performing hydrodelineation to create a mechanical cushion of the epinucleus [2, 7, 13, 15, 16]. Inside‑out delineation technique can precisely delineate the central nucleus, provides superior control, reduces stress to the zonules, and precisely demarcates the central core of the nucleus [17, 18].

Recently, Ravindra et al. have published their technique of controlled hydrodissection and nuclear rotation which gave excellent results with no PCR in retrospective analysis of 24 cases of PPC in manual small-incision cataract surgery [14].

An alternative technique was described using viscodissection in by Siatri and Moghimi and they also reported that there were no PCR in their series of 38 eyes [15]. They emphasize a caution in the presence of a white dot sign (or Daljeet Singh Sign), identified near the edge of the polar cataract [19].

Special modifications have been suggested to every step of cataract surgery in PPC including: incision, capsulorhexis hydrodelineation, rotation, division and fragment removal, epinucleus removal, cortex removal, posterior capsule vacuum polishing and also for femtosecond laser technology [3].

We practiced hydrodissection during PPC surgery for many years, following manual capsulorhexis (cases not included in the current study), and found this technique effective and relatively safe. Separation of the lens nucleus, as well as the posterior white plaque from the posterior capsule, is best done by a controlled fluid wave, prior to mechanical aspiration of the lens material. The posterior capsule is usually intact, even though thin and fragile. It can usually tolerate well a slow fluid (or viscoelastic) injection and once the nucleus is separated the risk is reduced to a minimal. In fact, in one case in this series and in one more case in previous experience, the posterior capsule broke by water-jet injection after removal of the nucleus and cortex, indicating a pathological extremely thin lens capsule. Viscodissection is a valid alternative, however the increase resistance of the viscous material may cause more pressure on the capsule than fluid and pose increased risk of capsule rupture.

The current series was different as the surgery was performed using femtosecond laser, The multiple air bubbles created by the laser-tissue interaction acts as "pneumo-dissection" and thus much less hydro-force is required to separate the nucleus from the capsule. Also, the continuous irrigation provided by an anterior chamber maintainer, reduced pressure fluctuation, hence reduce the risk of capsule tear. Phacoemulsification has been shown to be superior to manual extracapsular cataract extraction [16].

The combination of all of these components provides an efficient and relatively safe technique.

The use of femtosecond lasers during PPC surgery has already been reported [3, 20]. Vasavada et al. have described the technique of femtodelineation to enhance safety and reduce PCR rates in PPCs [11]. No hydrodissection or hydrodelineation procedure is performed in their method. Femtodelineation allows the creation of a mechanical cushion without using any hydro-procedure. It creates multiple nuclear layers or zones that act as shock absorbers during surgery. They prevent the transmission of mechanical forces as well as fluidic turbulence to the weakest part of the posterior capsule [11].

All 3 components of our suggested technique were previously reported, namely hydrodissection, femtosecond laser assisted cataract surgery (FLACS) and using anterior chamber maintainer (ACM). We present the significant advantages of combining the 3 components making this complex surgery safer and more reproducible. None of the methods is mandatory, especially FLACS which is not available in many places, and good results can be achieve using the traditional manual surgery, however in this cases too, hydrodissection is effective and valuable.

In recent years, the optical coherence tomography (OCT), allows the surgeons to visualize the integrity of the posterior capsule and can be useful in planning the surgical strategy and the additional risk of PCR during surgery. Kymionis et al. [21] have used OCT to judge the status of the posterior capsule before surgery and Chan et al. [22] used OCT imaging to grade PPCs and judge the presence or absence of PCR. [22] Titiyal et al. [20] reported the use of a microscope mounted intraoperative OCT system which give real‑time dynamic changes occurring during surgery to visualize integrity of the posterior capsule in PPCs. Bellucci et al. [23] employed real-time OCT to show that the posterior capsule receives a large amount of energy during the early phases of femtosecond laser lens fragmentation, being pushed behind by the very first laser bubbles.

In our experience with the integral OCT of the femtosecond laser the image indicates the location and extension of the lens opacity and the configuration of the affected posterior capsule. Nevertheless, unless the capsule is broken, the OCT image does not correlate with capsular integrity and even if the central capsule bulges posterior it is usually intact and separated from the opacified lens material.

It should be clearly emphasized that a preexisting tear in the posterior capsule is a completely different situation. Hydrodissection is then contraindicated, FLACS can be utilized onlr for the anterior capsulotomy and ACM can be used in low settings to minimize intraocular pressure. The discussion of the preferred surgical techniques is beyond the scope of the present paper.

## Limitations

The weaknesses of this study include the retrospective nature of the non-randomized study, and without randomization to reduce the impact of potential confounding patient or surgeon variables. Hydrodissection was not compared to viscodissection as an alternative technique.The number of cases is naturally relatively small, and more cases are required to prove the efficacy and safety of hydrodissection on PPC using modern technolohgies. Larger studies or pooled data may be able to provide better generalizability.

## Conclusion

This study shows that hydrodissection of the lens nucleus in eyes with PPC is indicated and effective provided there is no preexisting tear in the posterior capsule. Femtoseconf laser assisted cataract surgery in these eyes is safe and repeatable.

## Supplementary Information

Below is the link to the electronic supplementary material.

Video: Hydrodissection in an eye with posterior polar cataract using FLACS. Note the passage of fluid behind the central cataract and the free-floating white plaque. (MP4 37532 KB)

## Data Availability

No datasets were generated or analysed during the current study.
